# CRISPR/Cas Technologies and Their Applications in *Escherichia coli*

**DOI:** 10.3389/fbioe.2021.762676

**Published:** 2021-11-11

**Authors:** Huina Dong, Yali Cui, Dawei Zhang

**Affiliations:** ^1^ Tianjin Institute of Industrial Biotechnology, Chinese Academy of Sciences, Tianjin, China; ^2^ Key Laboratory of Systems Microbial Biotechnology, Chinese Academy of Sciences, Tianjin, China; ^3^ University of Chinese Academy of Sciences, Beijing, China

**Keywords:** *Escherichia coli*, CRISPR system, transcriptional regulation, knock-down, knock-in

## Abstract

The clustered regularly interspaced short palindromic repeats (CRISPR)/CRISPR-associated protein (Cas) systems have revolutionized genome editing and greatly promoted the development of biotechnology. However, these systems unfortunately have not been developed and applied in bacteria as extensively as in eukaryotic organism. Here, the research progress on the most widely used CRISPR/Cas tools and their applications in *Escherichia coli* is summarized. Genome editing based on homologous recombination, non-homologous DNA end-joining, transposons, and base editors are discussed. Finally, the state of the art of transcriptional regulation using CRISPRi is briefly reviewed. This review provides a useful reference for the application of CRISPR/Cas systems in other bacterial species.

## 1 Introduction

*Escherichia coli* is one of the most widely used cellular factories for the production of biofuels and bulk chemicals, such as ethanol, higher alcohols, fatty acids, amino acids, shikimate-derivatives, terpenoids, polyketides and polymer precursors such as 1,4-butanediol (Yang et al., 2021). Metabolic engineering for the production of these biochemicals requires extensive modulation of cellular metabolism for increased productivity. Genome editing requires efficient tools to perform time-saving sequential or multiplex manipulations.

Many gene-editing tools are available for *E. coli*, but all of them have specific strengths and shortcomings. Recombineering using double-stranded DNA (dsDNA) for genetic engineering often needs selectable markers, which should be eliminated in the following step for subsequent modifications (Datsenko and Wanner, 2000; Sharan et al., 2009). Compared with dsDNA, the efficiency of single-stranded DNA (ssDNA)-mediated recombineering is much higher, and it has been further developed into gene editing tools for multiplex editing such as Multiplex Automated Genome Engineering (MAGE) (Wang et al., 2009) and trackable multiplex recombineering (TRMR) (Warner et al., 2010). However, these methods are not suitable for multiple targeted gene insertions over 20 bp without selectable markers, and usually require robust high-throughput screening methods (Li et al., 2015).

**FIGURE 1:**
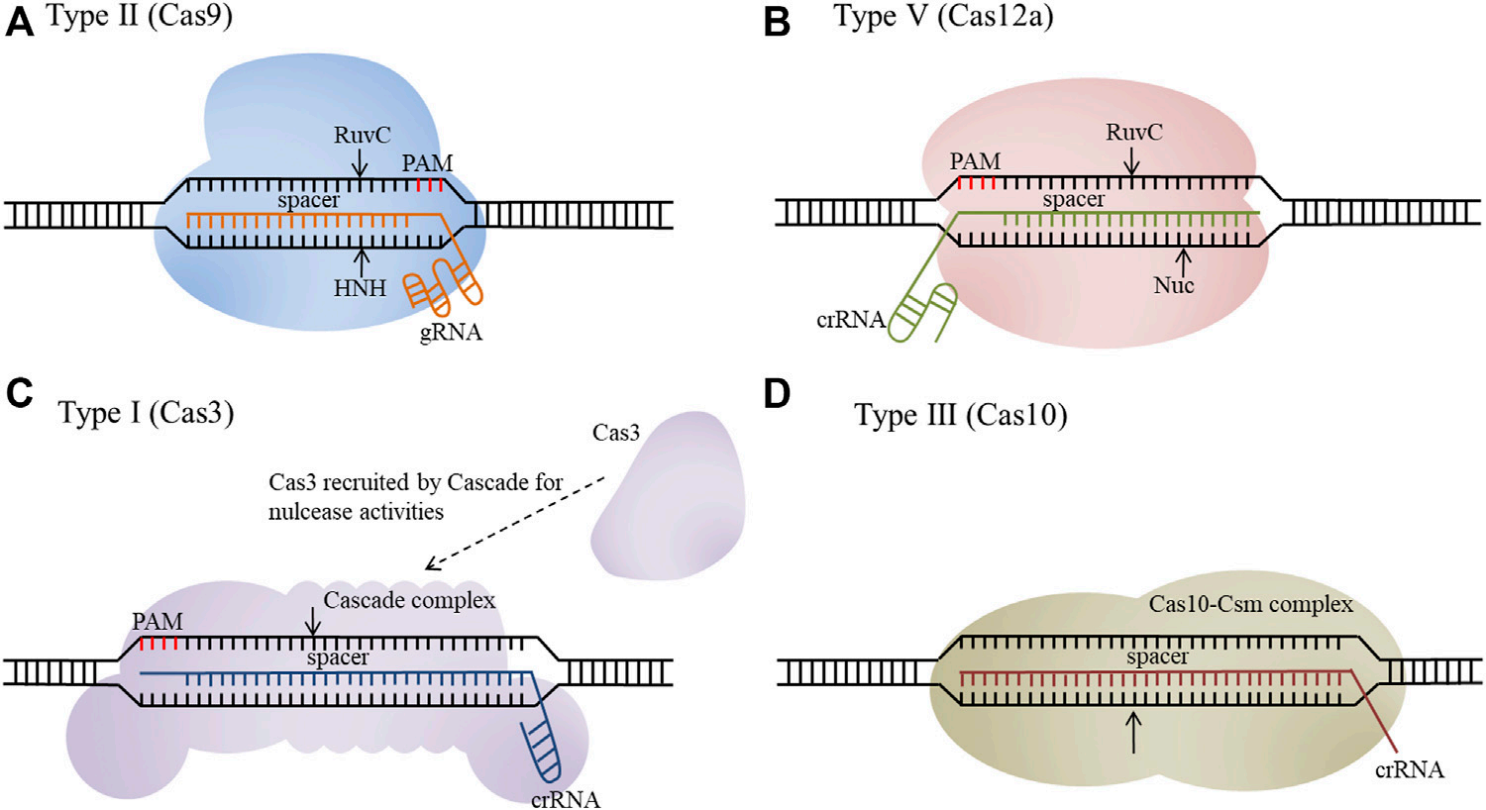
Schematic overview of CRISPR/Cas systems as genome engineering tools. **(A)** Type II (Cas9), **(B)** Type Ⅴ (Cas12a), **(C)** Type I (Cas3), **(D)** Type III (Cas10).

The recently developed clustered regularly interspaced short palindromic repeats (CRISPR)/CRISPR-associated protein (Cas) system is widely used for genetic engineering of *E. coli*, which has greatly promoted its application. A mature CRISPR RNA (crRNA) and *trans*-activating crRNA (tracrRNA) duplex (or a single synthetic guide RNA, sgRNA), or only a crRNA, guides the Cas nuclease(s) to cleave a target DNA sequence with a required protospacer adjacent motif (PAM) (Jiang et al., 2013). The mechanisms of different types of CRISPR systems have been summarized in our previously article (Liu et al., 2020). The CRISPR/Cas system continues to cut the target site until it is either successfully edited or the unedited cell dies, circumventing the need for selectable markers. According to the structure and function of the Cas protein, the CRISPR/Cas systems can be classified into two classes (class I and class II) and six types (type I∼VI) (Makarova et al., 2015). Class I includes types I, III, and IV, while class II includes types II, V, and VI (Mohanraju et al., 2016). Types I, II, and V recognize and cleave DNA, type VI can edit RNA, and type III edits both DNA and RNA (Wang et al., 2019). Since the structure of type II and type V systems is relatively simple, they are widely used in *E. coli*. The endogenous type I and type III systems have also been developed into efficient genome engineering tools for *E. coli* (Figure 1).

A series of reviews focusing on the mechanism and applications of CRISPR/Cas systems have been published (Arazoe et al., 2018; Choi and Lee, 2016; Liu et al., 2020; Pickar-Oliver and Gersbach, 2019; Zheng et al., 2020). Here, we focus on CRISPR/Cas systems as genetic tools for *E. coli.* We briefly introduce the mechanisms and the differences among the four types of CRISPR/Cas systems. At the same time, we summarize the development and application of CRISPR/Cas systems according to different types of genome editing and transcriptional regulation scenarios. We discuss the different deficiencies of current CRISPR/Cas technologies and offer possible directions for future development.

## 2 CRISPR/Cas-Mediated Genome Editing Based on Homologous Recombination

**FIGURE 2:**
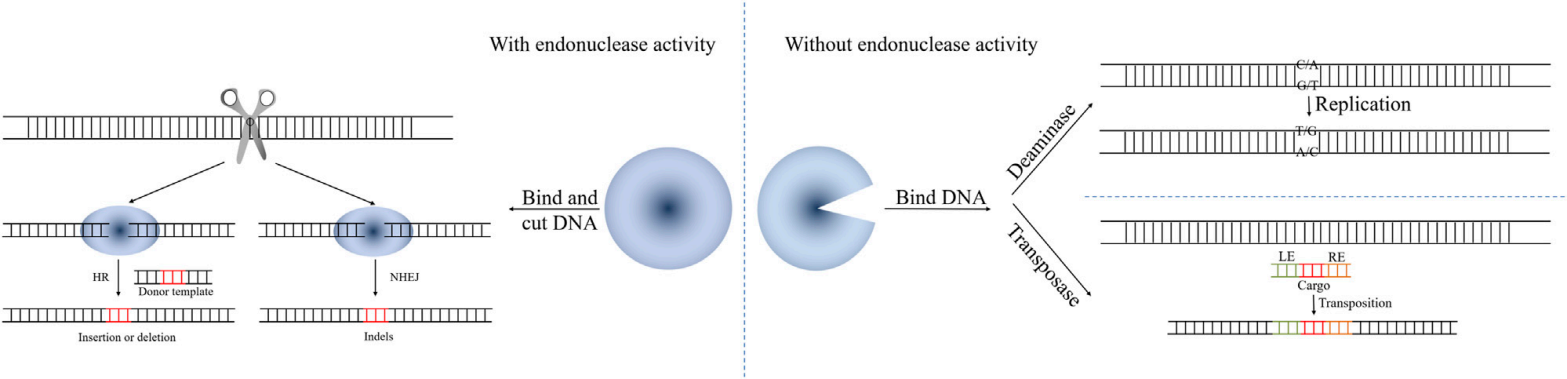
The mechanism of CRISPR/Cas-mediated genome editing based on HR, NHEJ, transposons (LE, RE, *cis*-acting **left** and **right** terminal sequences of Tn7-like transposons) and base editor.

CRISPR/Cas systems can produce targeted double-strand breaks (DSBs), which greatly increase the efficiency of homologous recombination (HR). As editing tools, they can introduce insertions, deletions and point mutations (Figure 2).

### 2.1 Type II CRISPR/Cas-Mediated Genome Editing Based on HR

The CRISPR/Cas9 genome editing system comprises the Cas9 protein, CRISPR RNA (crRNA) and *trans*-activating crRNA (tracrRNA). The currently most widely used Cas9 protein is derived from *Streptococcus pyogenes* (SpCas9). It contains 1368 amino acids, encompassing a REC domain with recognition function and a NUC domain with nuclease activity. A tracrRNA forms a crRNA duplex that directs the Cas9 protein to cut a target site with a proto-spacer adjacent motif (PAM). CRISPR/Cas9-mediated genome cutting kills cells that fail to be edited successfully, and therefore does not require markers for the selection of mutants (Li et al., 2015).

A CRISPR/Cas9 system containing Cas9, dual-RNAs (tracrRNA and crRNA), λ-Red proteins, and linear ssDNA for the introduction of precise mutations in the genome of *E. coli* achieved an efficiency of approximately 65% (Jiang et al., 2013). The major limitation of this method is the presence of escape mutants that avoid CRISPR-mediated cell death, which may result from the recombination of the repeat sequences flanking the target spacer. Subsequently, CRISPR/Cas9 systems containing Cas9, sgRNA, λ-Red proteins, and circular (Jiang et al., 2015) or linear donor DNA (Li et al., 2015) achieved efficiencies of up to 100%. These two systems contain an sgRNA plasmid curing system. Reisch *et al.* constructed a CRISPR/Cas9 system with an SsrA tag at the C-terminus of Cas9, also containing sgRNA, λ-Red proteins and linear donor DNA (Reisch and Prather, 2015). The SsrA tag is recognized by ClpP protease to degrade Cas9 that was present due to leaky transcription. The co-expression vectors for the four constructed CRISPR systems mentioned above and the λ-Red recombination system can be used to introduce gene knockouts, insertions or substitutions in the genome of *E. coli*, and notably do not need a selectable marker gene, thereby omitting the work needed for the deletion of the marker and greatly shortening the editing time.

In order to further simplify genome editing based on these systems, the Cas9 coding gene, sgRNA coding DNA, as well as the λ-Red recombination and editing template DNA were integrated into the same plasmid vector, and the gene editing cycle was shortened from about a week to 3 days (Zhao et al., 2016). The *recA* gene was then introduced into this one-plasmid system to simplify plasmid construction, as the addition of *recA* enabled the use of short homologous arms (41 bp) for successful genomic editing (Zhao et al., 2016). However, the editing efficiency with homologous arms with a length of 41 bp was only 13.8%, compared to nearly 100% with homologous arms of more than 300 bp.

To expand the application range of the CRISPR/Cas9 system, Zhao *et al.* developed a CRISPR/Cas9-assisted gRNA-free one-step (CAGO) technology in *E. coli*, which uses a linear donor DNA cassette and a pCAGO plasmid containing cas9, a sgRNA targeting universal N20 sequence, and the λ-Red system (Zhao et al., 2017). This technique does not require a specific sgRNA, but a universal N20PAM sequence with optimal targeting efficiency should be integrated into the *E. coli* chromosome by HR. This N20 sequence can then be targeted by CRISPR/Cas9 to generate a DSB and induce intra-chromosomal recombination. This technique can be used to edit a site with almost 100% efficiency in 2 days, edit PAM-free or CRISPR-tolerant regions with no off-target effects, and edit large areas of up to 100 kb with an efficiency of at least 75%. To further improve the efficiency of genome editing, CRMAGE technology based on multiplex automated genome engineering (MAGE), CRISPR/Cas9 and λ-Red recombination was established (Ronda et al., 2016). By repeatedly introducing synthetic ssDNA, MAGE can generate sequence diversity quickly and continuously at many chromosomal target positions in a large number of cells (Wang et al., 2009). The CRMAGE technology can introduce single point mutations to three target genes in *E. coli* with recombination efficiency between 96.5 and 99.7%, while the efficiency of traditional MAGE is only between 0.68 and 5.4%. The CRMAGE technology consists of two plasmids, one expressing λRed β protein and Cas9 protein, the other expressing an inducible sgRNA and a self-elimination system consisting of tracrRNA, which is combined with two crRNAs arranged in a natural CRISPR array. The β protein is co-expressed with *dam*, resulting in a *mutS* mutant phenotype, while *cas9* is expressed with *recX* to prevent the repair of double-strand breaks. The second plasmid is used for selection against wild-type sequences (Ryu et al., 2018).

Furthermore, to enable the deletion of large DNA fragments, a three-plasmid approach combining Cas9, tracrRNA, crRNA, λ-Red proteins, and linear dsDNA was developed (Pyne et al., 2015). This approach can induce DNA deletions of up to 19.4 kb and insert a heterologous DNA fragment of up to 3 kb. Then, a single-step integration strategy was developed by combining Cas9, sgRNA, λ-Red proteins, and linear or circular dsDNA containing a PAM mutation in the homology arm to ensure immunity of repaired cells (Bassalo et al., 2016). The integration efficiency of a 10-kb construct used to implement isobutanol production was above 50%. Chung *et al.* described a similar strategy based on a combination of Cas9, tracrRNA, crRNA, λ-Red proteins, and linear dsDNA containing 50 bp homology arms (Chung et al., 2017). The integration efficiency of a 7-kb foreign DNA exceeded 60%. The above three strategies use common recombineering methods based on homologous DNA sequences to overcome the size limit of integration. In *E. coli*, nicking Cas9^D10A^ can be used to form non-lethal single-stranded DNA breaks (SSBs) that still enable recombination between homologous sequences (Standage-Beier et al., 2015). A CRISPR/nCas9 system was constructed by combining nCas9 ^D10A^, targeting sgRNAs, and repeat sequences in the genome (Standage-Beier et al., 2015). The dual-targeting sgRNAs which target sequences adjacent to genomic repeats (e.g. the 1.2 kb IS5 elements) result in recombination across 36–97 kilobases and multiplex targeting enables the deletion of 133 kb. However, the current efficiency of this system is relatively low (<20%) and needs to be improved (Table 1).

### 2.2 Type V CRISPR/Cas-Mediated Genome Editing Based on HR

Type V CRISPR/Cas systems have a single RNA-guided RuvC-domain-containing effector, which is named Cas12 (also known as Cpf1). The RNA requirements of Cas12 effectors of different subtypes of Type V systems are listed in Table 2.

**TABLE 1:** Comparison of genome editing methods based on the CRISPR/Cas9 system.

Components						References
RNA-protein complex	Recombination system	Donor DNA	Length of homologous arms	Editing efficiency at a single locus	At multiple loci simultaneously		
dual-RNA:Cas9	λ Red	Linear (PCR products or oligonucleotides)	not mentioned	65%	Not tested	Jiang et al., (2013)
sgRNA:Cas9	λ Red	Circular (plasmids)	250–550 bp	Nearly 100%	About 50% for 3 point mutations	Jiang et al., (2015)^a^
sgRNA:Cas9	λ Red	Linear (PCR products or oligonucleotides)	300–500 bp	Nearly 100%	About 20% for 3 point mutations	Li et al., (2015)^a^
sgRNA:Cas9	λ Red	Linear (PCR products or oligonucleotides)	73-148 bp	Above 85%	Not tested	Reisch and Prather, (2015)
sgRNA:Cas9	λ Red + recA	Circular (plasmids)	41-300 bp	13.8 ± 7.9% (41 bp homologous arms) 100% (more than 300 bp homologous arms)	Not tested	Zhao et al. (2016)
sgRNA:Cas9	λ Red	Linear (PCR products or oligonucleotides)	about 500 bp	Nearly 100% (short fragment), 75% (about 100 kb)	Not tested	Zhao et al. (2017)
dual-RNA:Cas9；sgRNA:Cas9	λ/Red β-protein	Linear (PCR products or oligonucleotides)	70 bp	Nearly 100%	100% for 2 mutations	Ronda et al. (2016)
dual-RNA:Cas9	λ Red	Linear (PCR products or oligonucleotides)	40 bp	60% (deletion of 19.4 kb); nearly 60% (insertion of 3 kb)	Not tested	Pyne et al. (2015)
sgRNA:Cas9	λ Red	Circular (plasmids) or Linear (PCR products or oligonucleotides)	50-600 bp	70–100%	Not tested	Bassalo et al. (2016)
dual-RNA:Cas9	λ Red	Linear (PCR products)	50 bp	nearly 100% (deletion of short fragment); above 60% (deletion of 7 kb)	Not tested	Chung et al. (2017)
sgRNA: nCas9 D10A	Local-recombination system	Not required	repeat sequences, e.g. 1.2 Kb IS5 elements	<20%	Not tested	Standage-Beier et al. (2015)

a Indicates that this method includes a plasmid curing system.

Note: A specifically designed sgRNA can improve the editing efficiency (Hiranniramol et al., 2020).

**TABLE 2:** Type V Effectors^a^.

Effector	Subtype	Other names	Length	Target	tracrRNA	Cut type	PAM	References
Cas12a	V-A	Cpf1	1300 aa	dsDNA	No	staggered, 5 nt overhangs	5′ T-rich PAM	Yan et al. (2017)
Cas12b	V-B	C2c1	1129 aa	dsDNA	Yes	staggered, 7 nt overhangs	5′ T-rich PAM	Teng et al. (2018)
Cas12c	V-C	C2c3	1209-1330 aa	dsDNA	Yes	Flat	5′ TN PAM	Yan et al. (2019)
Cas12d	V-D	CasY	Approximately 1200 aa	dsDNA	No	—	5′ Y-rich PAM	Burstein et al. (2017)
Cas12e	V-E	CasX	980 aa	dsDNA	Yes	staggered, 10-11 nt overhangs	5′ TA PAM	Burstein et al. (2017)
Cas14a	V-F	—	529 aa	ssDNA	Yes	—	Not required	Harrington et al. (2018)
Cas12g	V-G	—	720-830 aa	ssDNA or ssRNA	Yes	—	—	Yan et al. (2019)
Cas12h	V-H	—	870-924 aa	dsDNA	No	flat	5′ TTN PAM	Yan et al. (2019)
Cas12i	V-I	—	1033-1093 aa	dsDNA	No	flat	5′ TTN PAM	Yan et al. (2019)

a Uncharacterized types, including V-U1 (C2c4), V-U2 (C2c8), V-U3 (C2c10), and V-U4 (C2c9), were omitted.

Cas12a has been extensively characterized both structurally and functionally, and was the earliest to be used in bacteria among the Type V effectors. A CRISPR/Cas12a system containing Cas12a, λ-Red proteins, crRNA, and linear donor DNA was constructed to introduce precise mutations in the genome of *E. coli* (Yan et al., 2017). The Cas12a protein was derived from *Francisella novicida* and uses a 5′-YTN-3′ PAM sequence. Compared with Cas9, the smaller Cas12a can reduce the metabolic burden of the host cell, and it is easier for researchers to deal with the corresponding material (e.g., in plasmid construction, electroporation, etc.). Then, a more efficient system was constructed by codon-optimizing the *FnCas12a* gene in the above system, and the donor DNA was placed on a plasmid rather than introduced as linear DNA (Ao et al., 2018). This method can insert up to 3 heterologous genes in one round of recombination, and the editing efficiency is about 20%. However, the editing efficiency of a single locus reached nearly 100%, compared to only approximately 50% before the optimization.

Cas12e from Deltaproteobacteria (DpbCas12e), also known as CasX, was identified as a new RNA-directed DNA endonuclease that uses a specific structure to cleave the target DNA (Burstein et al., 2017; Liu et al., 2019). DpbCas12e was shown to be active in *E. coli* when it was co-expressed with its sgRNA. The activity levels of DpbCas12e in manipulating the *E. coli* genome were only slightly lower than that of SpCas9 (Liu et al., 2019). The small size of Cas12e (<1,000 amino acids), low *trans*-cleavage activity, and its non-pathogenic origin offer important advantages for genome editing over Cas9 and Cas12a.

In addition to Cas12e, Cas12d (formerly CasY) has also been demonstrated to have RNA-dependent DNA interference activity in *E. coli* (Burstein et al., 2017). Recently, Cas12c, Cas12g, Cas12h, and Cas12i proteins were also characterized, and *in vivo* screening was performed in *E. coli* (Yan et al., 2019). Cas12c (also known as C2c3), with its crRNA and tracrRNA, as well as Cas12h or Cas12i with their own crRNAs, showed RNA-directed dsDNA interference activity. Cas12i showed significantly different efficiencies in the cleavage of complementary and non-complementary strands in the crRNA spacer region. Cas12g is a crRNA- and tracrRNA-directed RNase with collateral RNase and single-strand DNase activity. Cas14a was demonstrated to be a nonspecific ssDNA-targeting CRISPR endonuclease that does not require a PAM for its activation (Harrington et al., 2018). Although the CRISPR/Cas12d/e/c/h/i system has not been used to manipulate the genome of *E. coli*, it has potential to be developed into another general-purpose CRISPR/Cas system.

As another editing tool from the type V CRISPR/Cas family, the application of CRISPR/Cas12b, also known as CRISPR/C2c1, has not been reported in bacteria, but it has been reported in mammalian cells (Teng et al., 2018), where it enabled accurate single- and multigene editing. The Cas12b used in this system was from *Alicyclobacillus acidiphilus* (AaCas12b), and enabled robust genome editing with an sgRNA. Furthermore, the CRISPR/Cas12b system showed lower off-target effects in eukaryotes, along with a specificity for T-rich PAM sequences (L. Liu et al., 2017). Thus, the CRISPR/Cas12b system might be a good editing tool for bacteria.

Many newly discovered Cas12 effectors are still in the initial stages of characterization. However, the functional diversity of different type V CRISPR/Cas systems expands the CRISPR toolbox and is expected to be applied in prokaryotes such as *E. coli*.

### 2.3 Type I CRISPR/Cas-Mediated Genome Editing Based on HR

The Cas3 protein is a hallmark of type I CRISPR/Cas systems. The type I CRISPR/Cas systems are further divided into seven subtypes: I-A to I-F, and I-U, according to different subtype hallmarks. Type I-B (Cheng et al., 2017; Walker et al., 2020; J.; Zhang et al., 2018), type I-E (Hidalgo-Cantabrana et al., 2019) and type I-F (Zheng et al., 2019) systems combined with HR have been developed as genome editing tools for some bacteria. The endogenous I-E CRISPR/Cas system of *E. coli* has not yet been developed as a genome editing tool. The heterologously synthesized *Methanococcus maripaludis* type I-B system can protect *E. coli* against phage λ infection (Richter et al., 2017), highlighting a potential for genome editing using this type I-B system and HR in *E. coli*.

### 2.4 Type III CRISPR/Cas-Mediated Genome Editing Based on HR

The Cas10 protein is a hallmark of type III CRISPR/Cas systems. The type III CRISPR/Cas systems are further divided into four subtypes designated A-D. The interference target of the III-A subtype is DNA, while the target of III-B is RNA. Thus, the subtype III-A systems have the ability to edit the genome.

The *Staphylococcus aureus* type III-A system can induce large-scale genomic deletions and insertions, and its targeting activity is based on the complementarity between the crRNA and the protospacer sequence (Guan et al., 2017). The III-A CRISPR/Cas modules from *Lactococcus lactis*, *Staphylococcus epidermidis* and *Streptococcus thermophilus* were heterologously expressed in *E. coli*. It was found that the expression of these modules specifically eliminated an invasive plasmid recognized by the crRNA, which provides a new direction for the study of the III-A CRISPR/Cas systems in *E. coli* (Ichikawa et al., 2017) as these systems combined with HR may also be developed as genome editing tools in *E. coli.*

## 3 CRISPR/Cas-Mediated Genome Editing Based on Non-Homologous End-Joining

While CRISPR/Cas-mediated genome editing based on HR has been widely applied in *E. coli*, genome editing based on error-prone non-homologous DNA end-joining (NHEJ) has been widely applied in eukaryotes. NHEJ often produces small insertion and/or deletion (indel) mutations at the connection site, which can result in a frame-shift mutation of the target gene (Cui et al., 2019). The mechanism of CRISPR/Cas-mediated genome editing based on NHEJ is shown in Figure 2. However, most prokaryotic cells lack the NHEJ system, including *E. coli*.

Su *et al.* described a system based on CRISPR/Cas9-assisted NHEJ for the rapid and efficient inactivation of gene(s) and deletion of large chromosomal fragments in *E. coli* (Su et al., 2016). This strategy uses the NHEJ factors (ligase D and Ku protein) of *Mycobacterium tuberculosis* H37Rv and avoids the use of selection markers or a homologous DNA template. Zheng *et al.* tested three bacterial NHEJ systems, from organisms including *Mycobacterium smegmatis* (Msm), *M. tuberculosis* (Mtb) and *Bacillus subtilis*, in *E. coli* (Zheng et al., 2017). The most efficient system was MsmNHEJ, which was much more efficient than MtbNHEJ in the repair of DSBs (up to 45 times). The authors successfully deleted two large DNA fragments in *E. coli*. Furthermore, the efficiency of the strategy could be increased using phage T4 DNA ligase rather than ligase D and Ku protein (Su et al., 2019).

CRISPR/Cas9-assisted native end-joining editing (CNEE) was developed to delete segments of up to 83 kb or inactivate individual genes (Huang et al., 2019). In this method, a ligase D and Ku-independent *E. coli* native end-joining (ENEJ) system, also known as the alternative end-joining (A-EJ) system, was used to repair DSBs produced by Cas9. This CNEE depends on the RecBCD complex, and does not require highly competent cells with a high transformation rate, which reduces the difficulty of editing wild-type strains.

The CRISPR/Cas9-assisted NHEJ or CNEE system can be used in various strains, especially industrial strains with low HR efficiency. It plays an important role in functional genomic research, including the engineering of genes and genomes. The low DNA repair efficiency may be mainly caused by the low expression of NHEJ factors from the single gene copy on the chromosome, which may hinder the wider use of NHEJ.

### 4 CRISPR/Cas-Mediated Genome Editing Based on Transposons

As has been described in the preceding chapters, CRISPR/Cas nucleases are powerful tools for genetic manipulation. Although it is possible to achieve precise integration of foreign DNA after Cas-induced DNA cleavage through HR, the process has a low efficiency that greatly depends on the individual bacterial strain. Recently, transposon-based CRISPR genome editing technologies have been proposed to efficiently and accurately integrate mobile genetic elements into a specific site, in a manner that is not dependent on the induction of breaks in the DNA, which makes it much less likely to kill the cell. These technologies extended the understanding of the functional diversity of the CRISPR/Cas systems and enabled the establishment of methods for precise DNA insertion (Figure 2).

### 4.1 Type V CRISPR/Cas-Mediated Genome Editing Based on Transposons

A CRISPR-associated transposase from the cyanobacterium *Scytonema hofmannii* (ShCAST) was characterized by the team of Feng Zhang (Strecker et al., 2019). The ShCAST protein consists of Tn7-like transposase subunits (TnsB, TnsC and TniQ) and Cas12k, which are encoded by a gene cluster. TnsB is the nuclease that cleaves DNA, and TnsC is an ATPase that provide energy. TniQ and Cas12k identify targeted sites, and recruit TnsC to the site. Due to the interaction between TnsB and TnsC, TnsB cleaves the cargo gene, binds it to the site and inserts it 60–66 bp downstream of the PAM sequences. Researchers applied this system in *E. coli* and successfully inserted DNA fragments of 500 bp to 10 kb at locations 60–66 bp downstream of the PAM sequence with an efficiency of up to 80% without positive selection.

### 4.2 Type I CRISPR/Cas-Mediated Genome Editing Based on Transposons

In the same year, a CRISPR-associated transposase from *Vibrio cholerae* was characterized by the team of Samuel H. Sternberg and used in *E. coli* MG1655 (Klompe et al., 2019). The cascade complex, which is composed of Cas6, Cas7, and Cas8, binds directly to the TniQ protein to guide the transposon to the target in the genome. TnsA and TnsB promote the transposition reaction and TnsC provides energy. This insertion is highly specific, and the transposon was accurately and completely delivered to 25 different target sites in the bacterial genome.

## 5 CRISPR/Cas-Mediated Genome Editing Using a Base Editor

Unlike the nuclease-based CRISPR systems, base editors can perform genome editing without inducing DSBs (Komor et al., 2016; Murugan et al., 2017) (Figure 2). In principle, there are 12 possible base-to-base changes that may occur via individual or sequential use of transition (i.e., a purine-to-purine change or pyrimidine-to-pyrimidine change) or transversion (a purine-to-pyrimidine or pyrimidine-to-purine) editors. Researchers have established several base editors with different structures, catalytic activities, and potential modifications.

### 5.1 Type II CRISPR/Cas-Mediated Genome Editing Using a Base Editor

A cytosine-to-thymine base editor (or “CTBE,” “CBE”) converts a C:G base pair to a T:A base pair. Because the corresponding paired bases are also interchanged as a result of the conversion, this category of base editor may also be referred to as a guanine-to-adenine base editor (or “GABE”). A CTBE based on the fusion of CRISPR/dCas9 (catalytically dead Cas9^D10A,H840A^) and *Petromyzon marinus* cytosine deaminase (PmCDA1) was successfully established in *E. coli* (Banno et al., 2018). The mutation rate in the 15–20 base sequence recognized by the PAM was 61.7–95.1%. Then, the mutation efficiency was significantly improved by adding a uracil DNA glycosylase inhibitor (UGI) and a protein degradation label (LVA tag), which allowed the simultaneous editing of six different genes. An nCas9-cytidine deaminase (rat cytosine deaminase, rAPOBEC1) fusion protein was applied to convert targeted C-to-T bases in *E. coli* and *Brucella melitensis*, with efficiencies as high as 100% (Zheng et al., 2018).

An adenine-to-guanine base editor (“AGBE” or “ABE”) converts A:T to G:C. AGBE may also be referred to as a “TCBE.” An AGBE (ABE7.10) based on the fusion of CRISPR/nCas9, *E. coli* TadA adenine deaminase and evolved adenine deaminase (TadA^∗^) was successfully established in human cells (Gaudelli et al., 2017). The rapid protein evolution, engineering, and plasmid-based selection of TadA was performed in *E. coli*, and dCas9-TadA^∗^ was able to induce targeted A to G mutations (Gaudelli et al., 2017). ABE7.10 has been applied in various organisms in the past 2 years (Liu et al., 2018; Ryu et al., 2018). David R. Liu *et al.* (Richter et al., 2020) obtained an ABE8e system by evolving the deaminase component of ABE7.10 using phage-assisted non-continuous and continuous evolution (PANCE and PACE) in *E. coli.* ABE8e exhibited a dramatic increase in deamination kinetics and offers substantially improved editing efficiencies when paired with a variety of Cas orthologs. However, few studies investigated the use of ABE in prokaryotes.

Recently, Xiuqing Xin *et al.* compared the editing efficiency of nCas9-TadA^∗^ and dCas9-TadA^∗^ in *E. coli*, and found that the efficiency of dCas9-TadA^∗^ was very low while that of nCas9-TadA^∗^ was moderate (Xin et al., 2019). They then established a double-check base editing (DBE) tool by combining active Cas9 and nCas9-TadA^∗^, and the editing efficiency of ABE was improved significantly. A cytosine-to-adenine base editor (or “CABE”) converts a C:G base pair to a A:T nucleobase pair. CABE may also be referred to as a “TCBE.” A CABE consisting of a Cas9 nickase, a cytidine deaminase (PmCDA1) and the native *E. coli* uracil-DNA glycosylase (Ung) was constructed in *E. coli* (Zhao et al., 2020). Ung excises the uracil base created by PmCDA1, forming an apurinic/apyrimidinic (AP) site that initiates the DNA repair process. This system converts C to A with an average editing specificity of 93.8 ± 4.8% and editing efficiency of 87.2 ± 6.9%. However, when PmCDA1 in this system was replaced with rat APOBEC1, C-to-G conversions were observed in mammalian cells (M. F. Richter et al., 2020; Zhao et al., 2020). This means that C-to-G base editors (or “CGBE,” also referred to as a “TCBE”) were constructed in mammalian cells.

A universal base editor (prime editor or PE) was constructed by fusing a reverse transcriptase (RT) with an RNA-programmable nickase and a prime editing extended guide RNA (pegRNA) to achieve all 12 types of point mutations (Xin et al., 2019). This system exploits a brand-new way of genome editing and could work in yeast and human cells. However, the editing efficiency of PE was lower than that of other base-editing techniques and additional aspects of this systems also need to be improved for a more mature method, such as assessing off-target prime editing in a genome-wide manner.

### 5.2 Type V CRISPR/Cas-Mediated Genome Editing Using a Base Editor

G/C-rich PAM sequences limit the targeting range of the Cas9-fused base editor. Consequently, dLbCas12a-BE0 was constructed by fusing rat APOBEC1 and UGI to a catalytically inactive *L. bacterium* Cas12a (dLb-Cas12a) that can avoid the limitation of G/C-rich PAM sequences. The editing efficiency of dLbCas12a-BE0 was 44–74% in *E. coli* (X. Li et al., 2018)*.*

## 6 CRISPR/Cas-Mediated Transcriptional Regulation

RNA interference (RNAi) and engineered DNA-binding proteins such as zinc finger or transcription-activator-like effector (TALE) proteins were used as powerful tools for transcriptional regulation. However, RNAi sometimes exhibits significant off-target effects and toxicity, while the DNA-binding proteins cannot be used to regulate multiple genes simultaneously (Qi et al., 2013).

The CRISPR system provides a powerful platform for the simultaneous regulation of multiple targeted genes, enabling large-scale genetic regulation. There are diverse CRISPR systems in different organisms, and several CRISPR interference (CRISPRi) systems have been developed to regulate gene expression in *E. coli*.

### 6.1 Type II CRISPR/Cas-Mediated Transcriptional Regulation

The CRISPR/dCas9 system is based on a catalytically inactivated Cas9, which is combined with a guide RNA. This system can be used to efficiently inhibit the expression of multiple genes simultaneously in *E. coli*, and the effects are reversible as both dCas9 and sgRNA were placed under the control of an inducible promoter (Qi et al., 2013). Furthermore, the regulation is highly specific, without off-target effects (Qi et al., 2013).

Numerous studies have used the CRISPRi system to fine-tune the biosynthetic pathways of *E. coli* and thereby increase the yield of target products, such as terpenoids (Kim et al., 2016), pinosylvin (Liang et al., 2016), anthocyanins (Cress et al., 2017), or malate (Gao et al., 2018).

However, high expression of dCas9 will induce abnormal cell morphology in *E. coli* by influencing cell division as well as the structure of the inner and outer membranes (Cho et al., 2018). Thus, regulating the expression level of dCas9 is critical for efficient use of the CRISPR/dCas9 system.

### 6.2 Type V CRISPR/Cas-Mediated Transcriptional Regulation

A CRISPR/dCas12a system for multiplex gene regulation in *E. coli* was constructed based on a modified Cas12a from *Acidaminococcus* sp., carrying the E993A mutation in the RuvC domain (X. Zhang et al., 2017)*.* The dCas12a lost the DNase activity against both strands of target DNA but maintained its RNase activity. Inhibition by the CRISPR/ddCas12a system was achieved by targeting specific sites using CRISPR RNAs (crRNAs), and was more effective than that achieved using the CRISPR/dCas9 system.

### 6.3 Type I CRISPR/Cas-Mediated Transcriptional Regulation

When the DNA degradation function of the I-E system was inactivated, the resulting mutant could be used as a DNA-binding molecule to implement CRISPR interference. A native CRISPRi system was constructed in *E. coli* by deleting the *cas3* gene and activating the expression of the cascade operon (*casABCDE* genes) using the constitutive promoter J23119. The metabolic flux from the central metabolic pathway to the PHB synthesis pathway was successfully redirected using this system (Chang et al., 2016). A similar modified *E. coli* type I-E system was constructed by deleting the *cas3* gene and placing the cascade operon under the control of the arabinose-inducible pBAD promoter. Using this system, six different genes could be targeted simultaneously, which resulted in improved 3-hydroxypropionic acid (3HP) production (Tarasava et al., 2018).

Endogenous CRISPR systems can be powerful tools for regulating metabolic pathways because they do not impose a high metabolic burden as is the case with type II or V systems.

## 7 Future Prospects

Due to its ease of cultivation and availability of genetic manipulation tools, *E. coli* is often used as a host for the production of fine and bulk chemicals via metabolic engineering. The emergence of CRISPR/Cas technology has provided *E. coli* with more convenient and efficient genetic manipulation tools, which further promoted its use as an ideal industrial production platform. However, the currently available CRISPR/Cas technology still has certain shortcomings. New methods with reduced off-target rates are emerging, such as the fusion of dCas9 with FokI protein, which provides a new perspective for research, and more details on the off-target activities have been summarized in a recent review (Li et al., 2019). By continuously mining new Cas proteins and modifying existing Cas proteins, the limitations of PAM can be avoided as much as possible. With the discovery of new homologs, additional Cas proteins are becoming available for RNA editing when temporary changes are desirable or when DNA editing is challenging, such as programmable RNA targeting using the subtype III-E effector Cas7-11 (Özcan et al., 2021) or compact RNA editors based on the newly identified and characterized Cas13bt (Kannan et al., 2021).

Diverse CRISPR/Cas systems have already enabled impressive applications in *E. coli*. However, many non-model bacteria still lack effective and efficient genome editing tools. The CRISPR-based tools for *E. coli* will further promote the development of genome editing tools for non-model microorganisms, and provide more options for the functional study of their genomes.

The future discovery and characterization of novel CRISPR/Cas systems will lead to further expansion of CRISPR-based tools. The combination of detailed molecular studies and the development of molecular tools could further enhance the CRISPR/Cas toolkit for bacteria, which will further promote its applications.
